# Identifying Allosteric Hotspots in *Mycobacterium tuberculosis* cAMP Receptor Protein through Structural Homology

**DOI:** 10.1021/acs.biochem.4c00723

**Published:** 2025-01-31

**Authors:** Stephen P. Dokas, Daniel K. Taylor, Lydia L. Good, Sanuja Mohanaraj, Rodrigo A. Maillard

**Affiliations:** † Department of Chemistry, Georgetown University, Washington, District of Columbia 20057, United States; ‡ Institute of Soft Matter Synthesis and Metrology, Georgetown University, Washington, District of Columbia 20057, United States

## Abstract

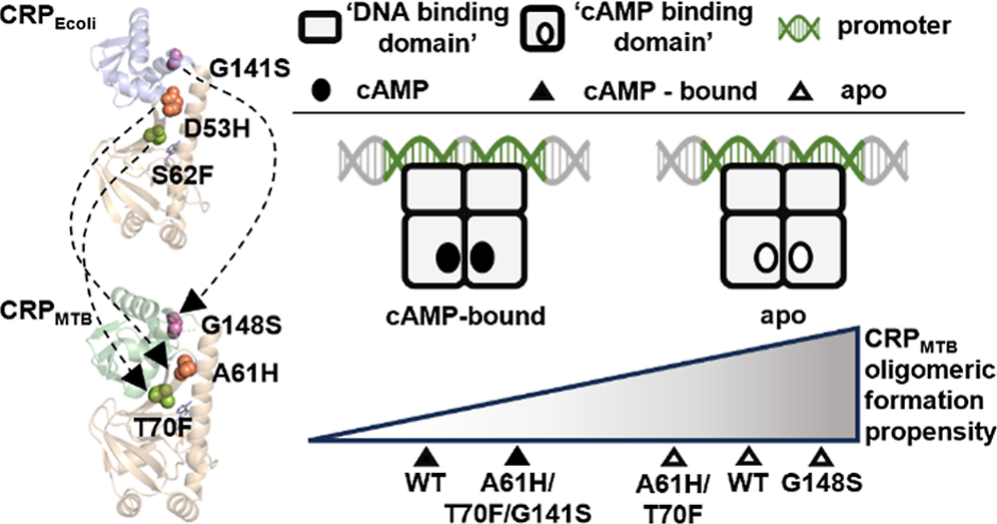

Understanding the mechanisms of allosteric regulation in response to second messengers is crucial for advancing basic and applied research. This study focuses on the differential allosteric regulation by the ubiquitous signaling molecule, cAMP, in the cAMP receptor protein from *Escherichia coli* (CRP_Ecoli_) and from *Mycobacterium tuberculosis* (CRP_MTB_). By introducing structurally homologous mutations from allosteric hotspots previously identified in CRP_Ecoli_ into CRP_MTB_ and examining their effects on protein solution structure, stability and function, we aimed to determine the factors contributing to their differential allosteric regulation. Our results demonstrate that the mutations did not significantly alter the overall fold, assembly and thermodynamic stability of CRP_MTB_, but had varying effects on cAMP binding affinity and cooperativity. Interestingly, the mutations had minimal impact on the specific binding of CRP_MTB_ to DNA promoter sites. However, we found that cAMP primarily reduces nonspecific CRP_MTB_–DNA complexes and that the mutants largely lose this ability. Furthermore, our experiments revealed that CRP_MTB_–DNA complexes serve as a nucleation point for additional binding of CRP_MTB_ proteins to form high-order oligomers with the DNA. Overall, our findings highlight the importance of both cAMP and DNA interactions in modulating the allosteric regulation of CRP_MTB_ and provide insights into the differential responses of CRP_Ecoli_ and CRP_MTB_ to cAMP.

## Introduction

Understanding how proteins respond to second messengers is a key area in both fundamental and practical scientific research. Second messengers often trigger protein responses across large distances within the protein structure, a process termed allostery. Initial models of allostery were based on the idea that proteins change in conformation upon ligand binding.^1^ However, current research acknowledges that allostery can also be the result of changes in the protein’s internal motions, leading to what is known as entropy-driven allostery.^2−4^ While the contributions of conformational changes and entropy to allostery are well documented,^5−8^ the mechanism by which structurally similar proteins exhibit diverse allosteric responses remains less understood. Addressing this matter is crucial for explaining how different species can use structurally conserved proteins in unique ways to adapt to their ecological niches.^9^ A notable example of such versatility is seen in the CRP-FNR (cAMP receptor protein–fumarate and nitrate reductase) family of transcription regulators.^9−11^ Despite all members of this family sharing common structural features, they display a remarkable variety of essential functions for organisms to adapt to environmental changes, influencing fitness and in the case of pathogens, virulence.

**Figure 1:**
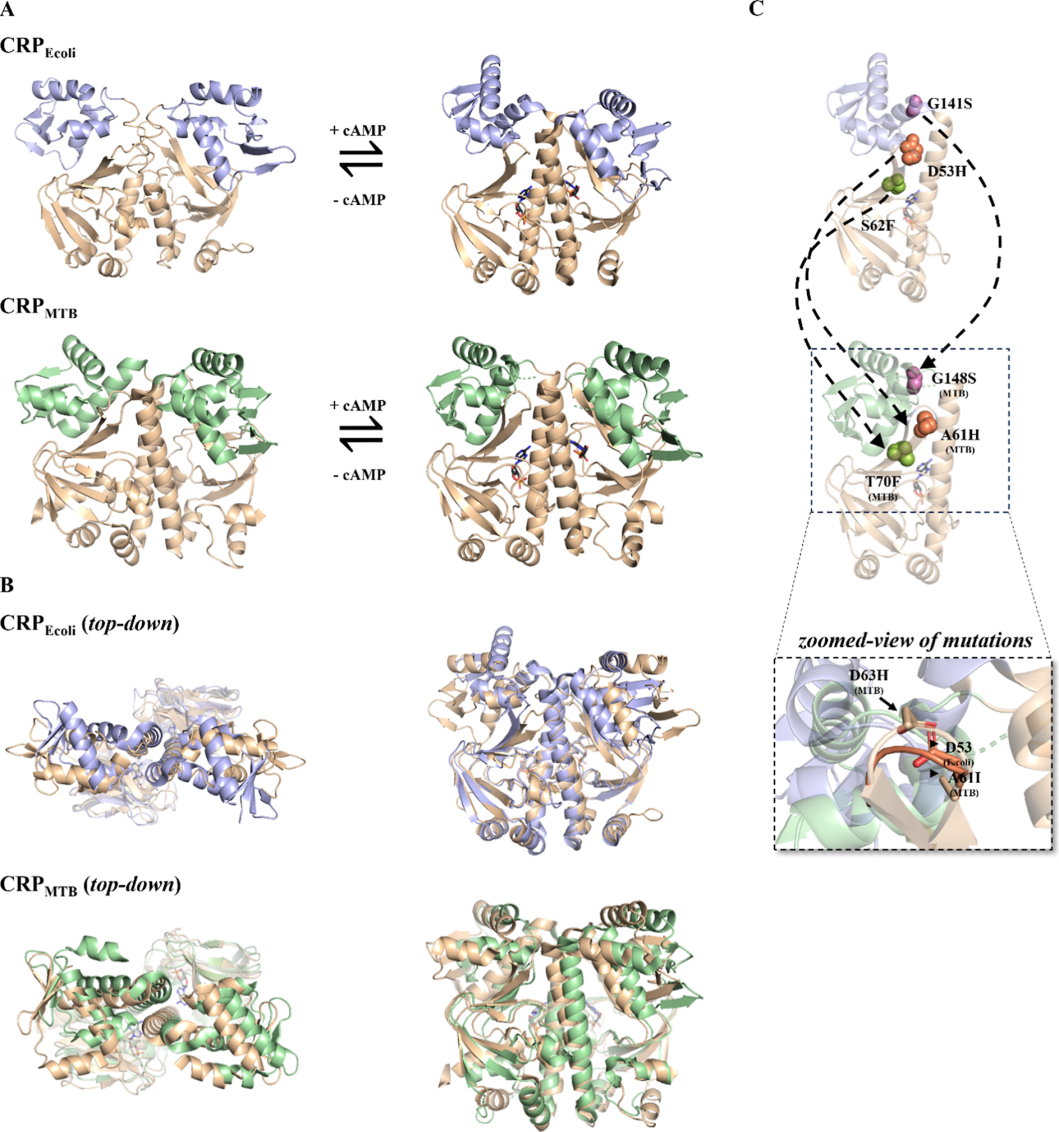
Crystal structures of CRP_Ecoli_ and CRP_MTB_ with structurally homologous mutations. (A) *apo*-CRP_Ecoli_ binds one cAMP molecule per CBD (tan) and undergoes reorganization of its central helices at the dimer interface and rotation of its DBD (blue) to become cAMP-bound CRP_Ecoli_. apo-CRP_MTB_ binds one cAMP molecule per CBD and its DBD (green) become more symmetric in the cAMP-bound conformation. (B) Top-down and side views of the alignments of the CBD of the apo (tan) and cAMP-bound (colored) structures; RMSD values for CRP_MTB_ are 0.27 Å for chain A and 0.31 Å for chain B; RMSD values for CRP_Ecoli_ are 1.33 Å for chain A and 1.31 Å for chain B. (C) structurally homologous residue positions between CRP_Ecoli_ and CRP_MTB_ monomers are indicated with dashed arrows and are respectively—D53H and A61H (orange spheres), S62F and T70F (green spheres), and G141S and G148S (purple spheres). A zoomed perspective of the alignment of CRP_Ecoli_ and CRP_MTB_ at “loop 3” shows the positions of the CRP_MTB_ mutants A61I and D63H; protein backbones are transparent. PDB codes are found in Materials and Methods.

CRP from *Escherichia coli* (CRP_Ecoli_) has been central to our understanding of protein allostery since its structure was first resolved in 1981.^12^ CRP_Ecoli_ binds two cAMP molecules at its N-terminal cyclic-nucleotide binding domains (CBD), triggering a structural rearrangement in the DNA binding domains (DBD) that allows it to tightly bind to DNA promoter sequences (Figure 1A).^12−15^ In contrast, apo CRP from *Mycobacterium tuberculosis* (CRP_MTB_), while structurally similar and sharing over half of its amino acid sequence with the apo-*E. coli* homologue,^16,17^ behaves quite differently. It binds to DNA with similar affinity whether or not cAMP is present.^17−19^ This difference is puzzling because CRP_MTB_ and cAMP are crucial for the MTB’s survival.^20−26^ Instead, in a recent study we found that CRP_MTB_ in the apo state forms nonspecific high-order oligomers on DNA that reversibly dissociate into a specific, one-to-one CRP_MTB_-DNA complex upon binding to cAMP. This suggests an allosteric regulation mechanism distinct from CRP_Ecoli_.^27^ In this study, we ask what drives the differential allosteric regulation by cAMP between CRP_Ecoli_ and CRP_MTB_.

A commonly used strategy to address this question is to introduce mutations at sites that are structurally similar across different proteins. This approach is particularly relevant here, as the root-mean-square deviation (RMSD) reveals that the cAMP-bound states of both CRP proteins exhibit remarkable similarity, with values of 0.84 and 0.72 Å in the CBD of chains A and B respectively, and 1.23 and 0.93 Å in the DBD of chains A and B. Prior research identified crucial residue positions in CRP_Ecoli_ where mutations can significantly affect its function.^28−30^ The mutations D53H and G141S enhance the protein’s affinity to both cAMP and DNA compared to the wildtype.^28,29^ Conversely, the mutation S62F reduces both cAMP and DNA binding affinity.^28,29^ The mutations at equivalent sites in CRP_MTB_ are A61H for D53H, T70F for S62F, and G148S for G141S (Figure 1B). Among these, A61 and D53 are noteworthy because of their distinct side chain properties. To explore the effect of these differences, we investigated two additional mutations in CRP_MTB_: A61I, to examine the impact of side chain flexibility, and D63H, to investigate the effect of side chain charge. D63 in CRP_MTB_ is located within a conserved loop where D53 resides in CRP_Ecoli_, near a hinge connecting the CBD and the DBD (Figure 1C, zoomed-in cartoon).

To understand how these mutations affect the structure and function of CRP_MTB_, we first examined the overall conformation and thermodynamic stability. We then measured how these mutated proteins affect cAMP binding and DNA interactions using the serC promoter.^31^ Lastly, we explored the mechanism by which CRP_MTB_ forms high-order oligomers with DNA by using two types of DNA sequences: one with a random sequence and another with a sequence that includes half of the serC promoter site.

Our study’s findings reveal that the mutations we analyzed do not significantly change how CRP_MTB_ binds to cAMP or to the serC promoter site. However, these mutations do disrupt the effect of cAMP on breaking down high-order CRP_MTB_ oligomers on DNA. Furthermore, our data suggest that the formation of these oligomers is largely due to individual CRP_MTB_ molecules binding to specific CRP_MTB_–DNA complexes. When we used a half-site variant of the serC promoter (i.e., half of its binding site scrambled) we find that cAMP did not prevent the formation of large CRP_MTB_ oligomers on DNA as seen for the full serC site, indicating that the role of the DNA goes beyond just serving as a scaffold for interactions with proteins.

Altogether, our results indicate that the structural similarity between two allosteric proteins from distantly related bacteria does not reliably predict their allosteric behavior nor identify allosteric hotspots involved in the response to molecular signals. We also discovered that the full DNA promoter sequence is necessary for cAMP to regulate the formation of CRP_MTB_ oligomers properly. This finding underscores the importance of the correct conformation of both CBD and the DBD in CRP_MTB_ for forming a stable complex that can regulate transcription.

## Materials and Methods

### CRP_MTB_ and CRP_Ecoli_ Structures

All PDB structures presented in this study were generated using the PyMol Molecular Graphics System (Version 2.0, Schrödinger, LLC). RMSD were calculated using PyMol’s “align” command. For domain-specific alignments, the following residue ranges were utilized: the CBD of CRP_MTB_ (residues 28–110) and CRP_Ecoli_ (residues 21–104), and the DBD of CRP_MTB_ (residues 145–215) and CRP_Ecoli_ (residues 139–209). Protein Data Bank IDs (PDBs) used for CRP_MTB_ apo and cAMP-bound states were 3D0S and 3I54 respectively, and in CRP_Ecoli_ the PDB codes for apo and cAMP-bound states were 2WC2 and 1G6N, respectively. The experimental conditions reported for these structures in the Protein Data Bank are as follows: 3D0S (2.00 Å, X-ray diffraction, vapor diffusion at pH 7.5 and 298 K.); 3I54 (2.20 Å, X-ray diffraction, vapor diffusion at pH 9.5 and 298 K with cAMP); 2WC2 (solution NMR 90% H_2_O/10% D_2_O at pH = 6.0 and 305 K); 1G6N (2.10 Å, X-ray diffraction, microdialysis at pH 7.5 and 298 K with cAMP).

### Cloning, Expression, and Purification of CRP_MTB_ Wildtype and Mutants

The wildtype DNA sequence of CRP from *M. tuberculosis* (CRP_MTB_) was used in this study (UniProtKB P9WMH3). PCR was used for CRP amplification (PfuUltra Polymerase from Agilent Technologies), which was flanked by NdeI and *Bam*HI restriction sites. The PCR product was digested with NdeI and *Bam*HI according to manufacturer protocol [New England Biolabs (NEB)]. A His-tag was fused to the final construct through insertion into a pET-3a expression vector (Addgene). CRP_MTB_ mutants [A61H, A61I, D63H, G148S, and T70F] were made following the QuikChange II Site-Directed Mutagenesis protocol (Agilent Technologies). All CRP_MTB_ proteins were expressed in *E. coli* strain T7 Express pLysS competent cells (NEB). Bacteria were grown in LB media overnight and protein expression was induced with 1 mM IPTG for 2 h. Bacterial pellets were resuspended in lysis buffer (20 mM Tris, 200 mM NaCl) at 10 mL/g of pellet wet weight with protease inhibitors (10 mM benzamidine, 0.4 mM AEBSF, 1 μM pepstatin, 1 μM leupeptin, 28 μM TPCK/TLCK, 10 μM IMBX, 1 mM PMSF). The bacterial solution was homogenized and lysed with a M-110P Microfluidizer at 10,000 psi (Microfluidics). The resultant lysate was centrifuged at 15,000 rpm for 45 min at 4 *C*° in a JA 25.50 rotor (Beckman Coulter). The supernatant was combined with His60 Ni Superflow Resin (Takara Bio) overnight and supplemented with 30 mM imidazole. The supernatant flow-through was collected the next day. The resin was washed twice with 40 mL of lysis buffer supplemented with 30 mM imidazole. Elutions were collected by lysis buffer supplemented with 500 mM imidazole. Elutions containing CRP_MTB_ were combined, ran through size-exclusion chromatography, and stored at −80 °C in storage buffer (50 mM HEPES, 150 mM KCl, 1 mM EDTA, pH 7.2). All proteins elute ∼89 mL, which is a volume consistent with CRP as a dimer. Protein concentration was determined at 280 nm (ε = 24,980 cm^–1^ M^–1^).

### Protein Solution Structure

The secondary structure of CRP_MTB_ was monitored by circular dichroism (195–260 nm) on an Aviv model 202–01 spectrometer. Intrinsic tryptophan fluorescence was monitored in CRP_MTB_ at λ_ex_ = 285 nm and λ_em_ = 335 nm on a PTI QM40 fluorimeter. Both measurements were at protein concentrations of 5 μM in storage buffer.

### Chemical Denaturation with Guanidine Hydrochloride (GdnHCl)

Global protein unfolding was monitored by changes in intrinsic fluorescence (λ_ex_ = 285 nm and λ_em_ = 335 nm) on a PTI QM40 fluorimeter and secondary structure unfolding was monitored by circular dichroism absorption at 222 nm on an Aviv model 202–01 spectrometer. In both sets of experiments, we used 5 μM of protein in a buffer containing 150 mM KCl, 50 mM HEPES, 1 mM EDTA pH 7.6. Three independent titrations were performed for each protein and corrected for buffer effects to the signal. Data was fitted according to the linear extrapolation method.^54^ For CRP_MTB_ wildtype and structurally homologous mutants the data was fitted to a two-state unfolding model^54^
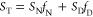
1where *S*_T_ is the total observed signal, *S*_N_ and *S*_D_ correspond to the native and denatured state signals, respectively, and *f*_N_ and *f*_D_ are the fractions of native and denatured protein, respectively. *f*_N_ and *f*_D_ are related to the equilibrium constant between folded and unfolded states
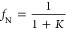
2
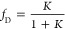
3where
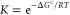
4and
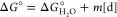
5 is the free energy of unfolding in the absence of denaturant, *m* is the *m* value or the slope of the linear dependence of Δ*G*° on denaturant concentration as described by the linear extrapolation method^54^ and [d] is the denaturant concentration. Combining eqs 1–5 yields the fitting equation
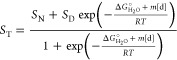
6

### cAMP Binding via ANS Fluorescence

Monitoring the fluorescent signal of the 8–anilino–1–napthalenesulfonic acid (ANS)–CRP_MTB_ complex (λ_ex_ = 350 nm and λ_em_ = 480 nm) reports on the binding of cAMP to CRP_MTB_. Solutions were prepared with 47.7 μM ANS and 3.6 μM (dimer) protein in ANS buffer (50 mM Tris; 50 mM KCl; 1 mM EDTA; pH 7.8) in a total volume of 800 μL. Data was taken on a PTI QM40 fluorimeter and at least three independent titrations were averaged and corrected for dilution and the interaction between ANS and cAMP alone. CRP_MTB_ wildtype and mutant data were fitted to a cooperative two-state binding model as described in Lanfranco et al.^32^ an independent two-site binding model, and a single-site binding model when no binding was detected for a second cAMP molecule. The cooperative model is shown in eq 7.
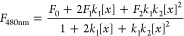
7where *F*_480nm_ is the observed signal; *F*_0_, *F*_1_, and *F*_2_ represent the fluorescent signal of the apo, singly bound, and doubly bound states of the protein, respectively; *k*_1_ and *k*_2_ corresponds to the microscopic binding affinity constants of the first and second cAMP, respectively, and *x* is the concentration of cAMP. In the independent binding model, *k*_2_ = *k*_1_ which assumes that the cAMP binding sites are not allosterically linked (i.e., no cooperativity). The ANS-based fluorescence data is normalized to the initial fluorescence value in the absence of cAMP.

### DNA Binding via Fluorescence Anisotropy

Measurements were collected with a PTI QM40 fluorimeter using a 32-bp serC promoter (5′-GCGCGTAGTGTGAACAAGCTCACATGCAAGCC-3′), covalently linked to a fluorescein molecule (IDTDNA), with a λ_ex_ = 480 nm and λ_em_ = 518 nm. The reaction mixture contained 3 nM of fluorescein-labeled DNA in total volume of 2 mL of DNA binding buffer (75 mM KCl, 50 mM HEPES, 1 mM EDTA, pH 7.6) and either 0 mM or 1 mM of cAMP. For stochiometric binding titrations we used the same experimental conditions except for labeled DNA at 200 nM. Data shown comes from at least three independent titrations and all data was baseline-corrected with the first experimental anisotropy value, and analyzed as described previously.^27,32,33^ The data was fitted according to eq 8
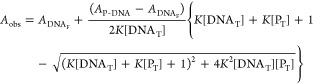
8*A*_obs_ is the observed anisotropy, *A*_DNAF_ and *A*_P-DNA_ are the anisotropy values for free DNA and protein–DNA complex, respectively, [DNA_T_] is the total DNA concentration, [*P*_T_] is the total protein concentration, and *K* represents the association constant for protein and DNA promoter sequences with (“*K*_DNA-cAMP_” in text) and without cAMP (”*K*_DNA_” in text).

The boundaries for forming high-order oligomers in the presence of 1 mM cAMP was determined as the lowest CRP concentration where the average anisotropy value significantly surpassed the threshold of specific DNA binding. This threshold was established by the fitting parameters from eq 8: *S*_F-cAMP_ + 2 × SD (Figure 4A and eq 8). The boundary for the formation of oligomers in the absence of cAMP was determined as the CRP concentration that intersects the linear extrapolation to the *y*-axis from the anisotropy value associated with [CRP] boundary with cAMP (Figure 4A). The apparent affinities (“*K*” = 1/[CRP]) associated with these boundaries were converted to Gibbs’s free energy values using eq 4. “Δ*G*_cAMP_” was determined by subtracting the free energy associated with the formation of oligomers in apo conditions from that of the free energy associated with the formation of oligomers with cAMP present (Supplementary Table S1).

## Results

### Solution Structure, Stability and cAMP Binding

**Figure 2:**
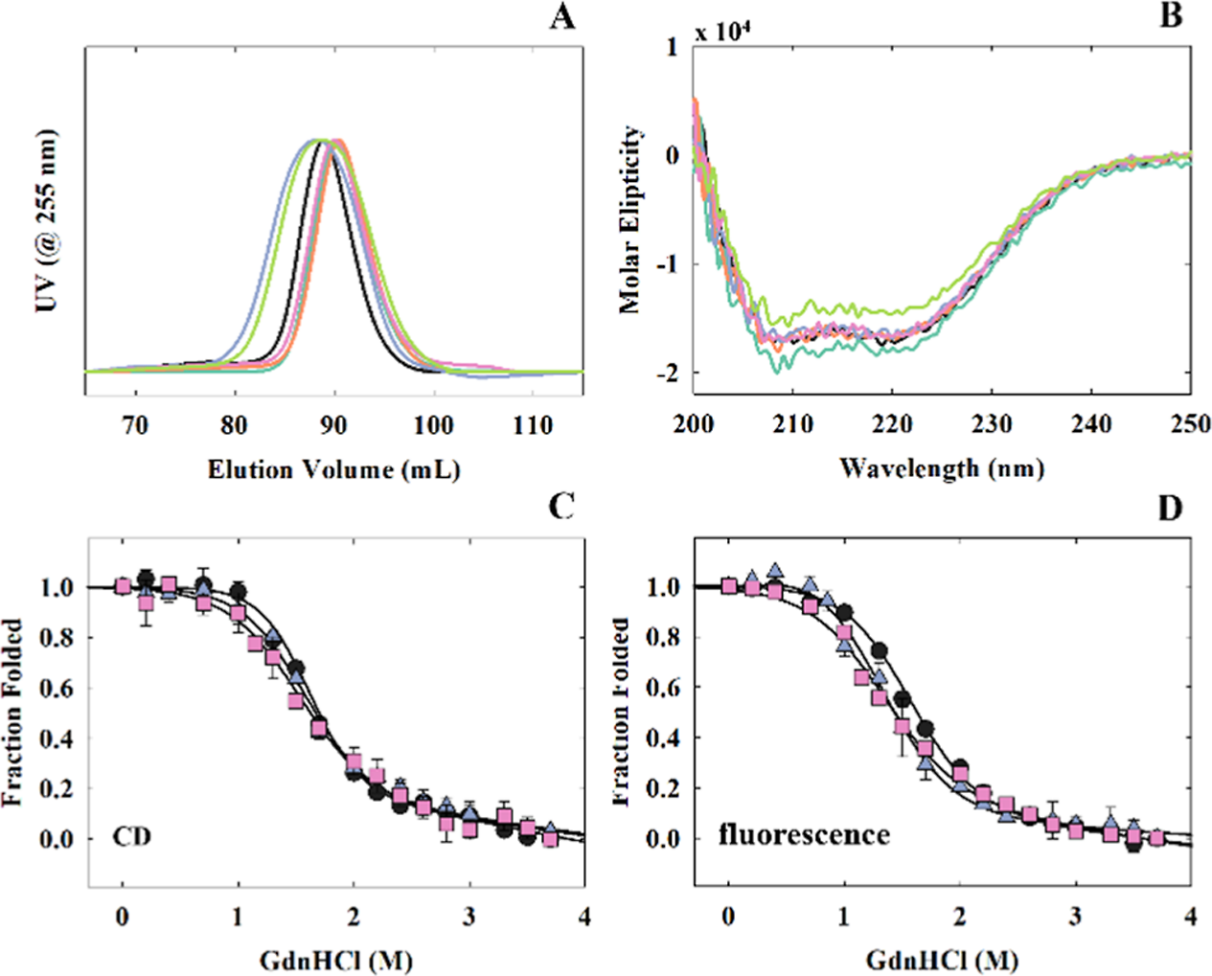
Biophysical characterization of CRP_MTB_ wildtype and mutants. (A) SEC elution profiles of CRP_MTB_ proteins. (B) native CD spectra of CRP_MTB_ proteins. Chemical denaturation of CRP_MTB_ wildtype, A61H, and G141S using guanidine-HCl measured by CD (C) (λ_EM._ = 222 nm) and intrinsic protein fluorescence (D) (λ_EX._ = 285 nm and λ_EM._ = 333 nm). Solid lines are the respective fits using a two-state unfolding model (eq 6). Key: wildtype (black), A61H (blue), A61I (teal), D63H (orange), T70F (green), G148S (pink).

We initially characterized the solution structure of CRP_MTB_ wildtype and its mutants using size-exclusion chromatography (SEC) and circular dichroism (CD) (Figure 2A,B). The mutant SEC elution profiles and CD spectra were similar to CRP_MTB_ wildtype (Figure 2A,B). We also evaluated whether the mutations disrupted the thermodynamic stability of CRP_MTB_ wildtype by monitoring changes in CD and fluorescence with varying concentrations of guanidine hydrochloride (GdnHCl) (Figure 2C,D). Both CRP_MTB_ wildtype and mutants underwent a two-state unfolding process, where only A61H and G148S displayed ∼30% lower unfolding free energies (Δ*G*°) and *m*-values compared to the wildtype (Table 1). Collectively, these data suggest that the mutations did not significantly alter the secondary or tertiary solution structure present in CRP_MTB_ wildtype.

**Table 1:** Thermodynamic Stability of CRP_MTB_ Wildtype and Mutants

	CD		fluorescence	
CRP_MTB_ protein^a^	Δ*G*°	*m*	Δ*G*°	*m*
wildtype	4.4 ± 0.2	–2.7 ± 0.1	3.1 ± 0.1	–2.0 ± 0.1
A61H	3.2 ± 0.2	–2.1 ± 0.1	2.5 ± 0.2	–2.0 ± 0.1
A61I	4.0 ± 0.3	–2.6 ± 0.2	2.9 ± 0.1	–2.1 ± 0.1
D63H	3.9 ± 0.3	–2.6 ± 0.2	2.8 ± 0.1	–1.9 ± 0.1
T70F	4.4 ± 0.3	–2.9 ± 0.2	2.9 ± 0.1	–2.0 ± 0.1
G148S	2.8 ± 0.2	–1.9 ± 0.1	2.4 ± 0.2	–1.8 ± 0.1

a CRP_MTB_, cAMP-receptor protein from *Mycobacterium tuberculosis*; Δ*G*°, free energy change; *m*, is the *m*-value. The error corresponds to the SD from fitted parameters using a two-state model as described in Materials and Methods. The units of Δ*G*° and *m* are kcal·mol^–1^ and kcal·mol^–1^·M^–1^, respectively.

**Figure 3:**
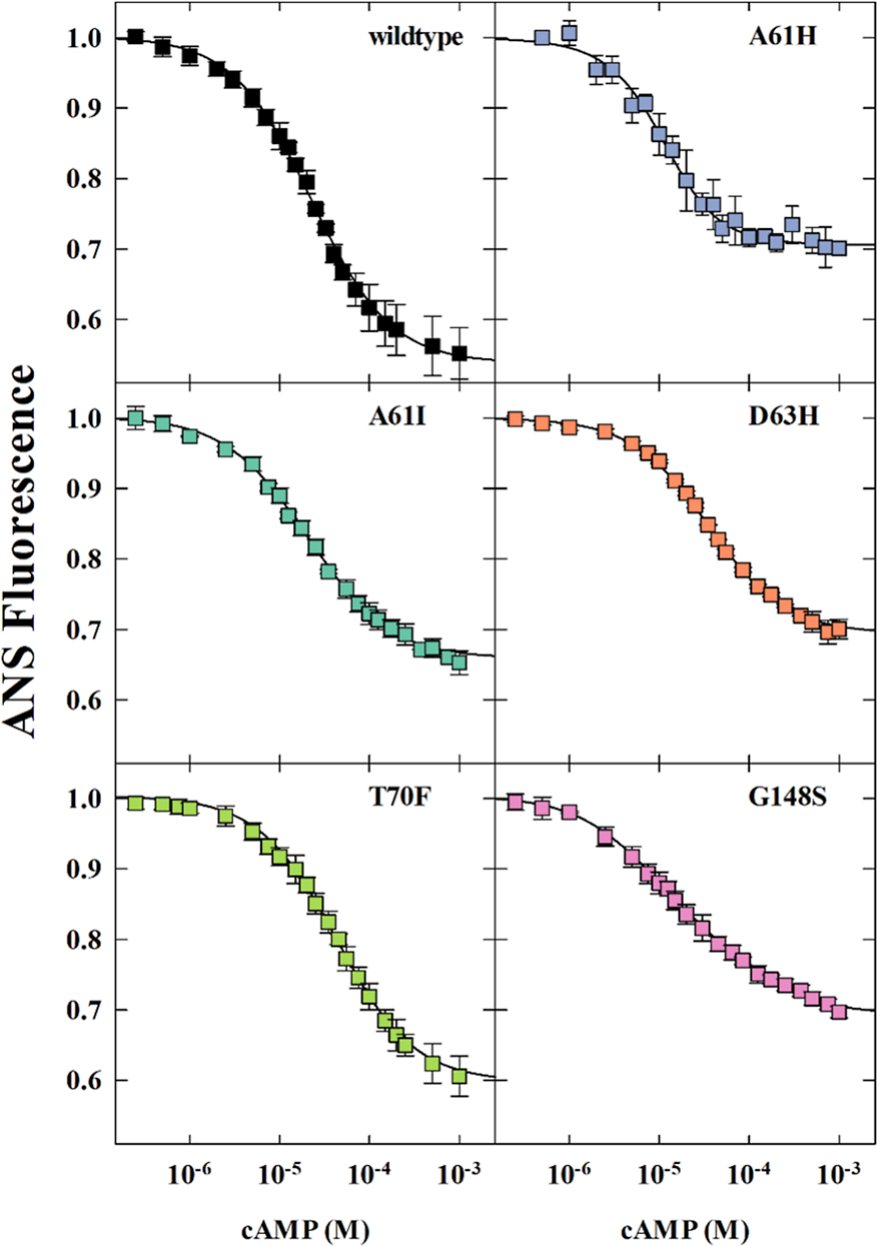
cAMP binding to CRP_MTB_ proteins via ANS fluorescence. Wildtype, A61H, A61I, and G148S isotherms were fitted to the two-site sequential binding model (eq 7). T70F and D63H isotherms were fitted to the two-site independent binding model (*k*_1_ = *k*_2_). Solid lines represent the fits for the respective CRP_MTB_ protein. Mutants are indicated for each isotherm.

We characterized the functional effects of each mutation on cAMP binding by measuring changes in the fluorescence of the reporter molecule 8-anilino-1-naphthalenesulfonic acid (ANS).^27,28,32^ Based on previous studies,^27^ we employed a two-binding site model to fit the binding isotherms, with *k*_1_ and *k*_2_ representing the first and second cAMP binding sites, respectively. For CRP_MTB_ wildtype we obtained *k*_1_ = 3.1 ± 0.4 M^–1^ and *k*_2_ = 2.4 ± 0.8 M^–1^, which is consistent with previous published work (Figure 3, Table 2).^27^ A61I displayed a modest increase in affinity for both cAMP binding sites, while G148S increased the cAMP-binding affinity only for the first site (Figure 3, Table 2). D63H and T70F slightly reduced the affinity for the first cAMP molecule, but not the second. Additionally, these two mutants exhibited identical *k*_1_ and *k*_2_ values, indicating a loss of cooperativity between the cAMP binding sites (Figure 3, Table 2). Interestingly, A61H was the only mutant with a significantly higher affinity for the second cAMP molecule, resulting in a positive cooperativity value of *c* = 4.8 ± 2.0 (Figure 3, Table 2).

**Table 2:** cAMP Binding Affinity for CRP_MTB_ Wildtype and Mutants

	cAMP-binding affinity and binding cooperativity^a^		
CRP_MTB_ protein	*k*_1_	*k*_2_	*c*
wildtype	3.1 ± 0.4	2.4 ± 0.8	0.8 ± 0.3
A61H	3.5 ± 0.9	16.9 ± 5.7	4.8 ± 2.0
A61I	7.2 ± 1.8	5.3 ± 0.9	0.7 ± 0.2
D63H	2.2 ± 0.3	2.2 ± 0.3	1.0 ± 0.2
T70F	2.2 ± 1.0	2.2 ± 1.0	1.0 ± 0.6
G148S	7.2 ± 0.8	2.1 ± 0.4	0.3 ± < 0.1

a *c*, cooperativity factor between cAMP-binding sites; CRP_MTB_, cAMP-receptor protein from *Mycobacterium tuberculosis*; *k*_1_, cAMP-binding affinity for the first cAMP binding site; *k*_2_, cAMP-binding affinity for the second cAMP-binding site. The error corresponds to the SD from fitted parameters as described in Materials and Methods. The units of *k*_1_ and *k*_2_ are 10^4^ M^–1^ and c = *k*_2_/*k*_1_.

### Allosteric Effect of cAMP in CRP_MTB_–DNA Interactions

**Figure 4:**
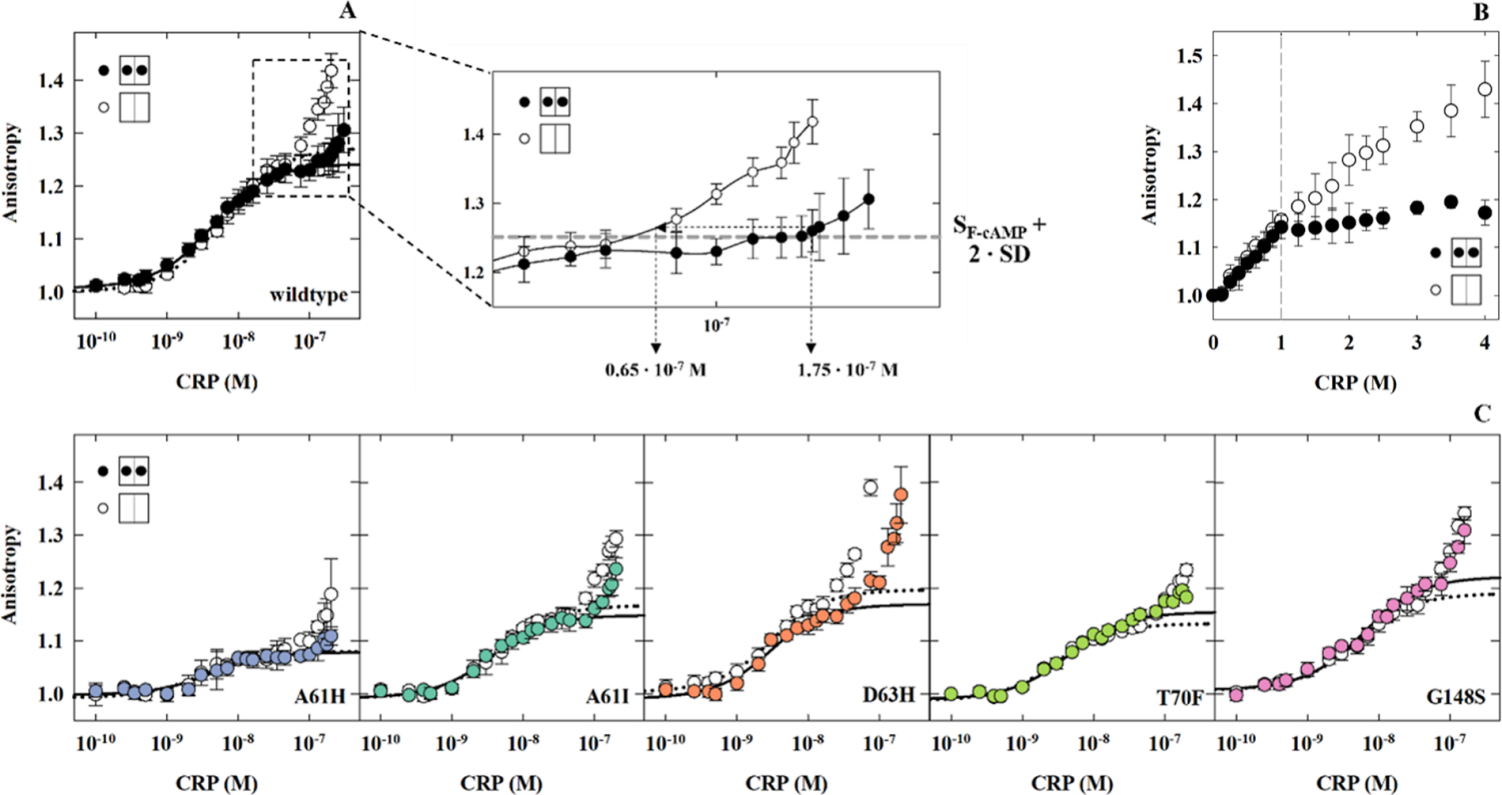
serC32-promoter binding to CRP_MTB_ proteins measured by fluorescence anisotropy. (A) serC32 binding data for CRP_MTB_ wildtype with and without cAMP, the zoomed window shows the determination of lower boundaries for the formation of oligomers on DNA based on fitting statistics from eq 8 (Materials and Methods); the lower boundaries serve to calculate Δ*G*_cAMP_ values (see Supplementary Table S1 for complete data). (B) Stochiometric binding data for CRP_MTB_ wildtype and the serC promoter. (C) serC32 binding data for CRP_MTB_ mutants with and without cAMP. Empty and filled circles indicate conditions with 0 and 1 mM cAMP, respectively. Dashed and solid lines represent the fits using eq 8 with and without cAMP, respectively. Mutants are indicated for each isotherm.

We employed fluorescence anisotropy to track the binding interactions between CRP_MTB_ and a fluorescein-labeled 32-bp DNA fragment containing the serC promoter. This analysis showed that the CRP_MTB_–DNA complex formation underwent two distinct binding transitions, as indicated by a biphasic change in the anisotropy signal (Figure 4A). The initial transition, occurring at CRP_MTB_ concentrations below 50 nM, corresponds to CRP_MTB_ interacting with the serC promoter sequence, resulting in a specific CRP_MTB_–DNA complex (Figure 4A). The subsequent transition, observed when CRP_MTB_ concentrations exceeded 100 nM, indicates the formation of high-order CRP_MTB_ oligomers on DNA.

In contrast to CRP_Ecoli_,^32,33^ analysis of the initial transition in Figure 4 indicates that the binding affinities are similar to or without cAMP (denoted as *K*_DNA-cAMP_ and *K*_DNA_, respectively). CRP_MTB_ wildtype showed *K*_DNA-cAMP_ = (2.81 ± 0.01) × 10^8^ M^–1^ and *K*_DNA_ = (1.87 ± 0.01) × 10^8^ M^–1^, in agreement with previous studies.^27^ However, the allosteric effect of cAMP in CRP_MTB_ becomes apparent during the second transition, at [CRP] > 100 nM. Here, high-order oligomers are more prominent and form at lower protein concentrations without the cyclic nucleotide. The presence of oligomers is further supported by stoichiometric binding experiments (Figure 4B; Materials and Methods). When cAMP is present, the anisotropy signal reaches a plateau at a one-to-one molar ratio of CRP to DNA, indicating the formation of a stable, one-to-one CRP_MTB_-DNA complex. In contrast, in the absence of cAMP, the anisotropy signal continues to rise as the molar ratio of CRP_MTB_ to DNA increases beyond one, indicating that free apo CRP_MTB_ proteins are binding to pre-existing specific protein–DNA complexes. We observed up to four CRP proteins bound to a single serC promoter. This molar ratio represents a lower limit because at higher protein concentrations these oligomeric complexes start to aggregate.

Given the unique response of CRP_MTB_ to cAMP binding, we aimed to identify the factors driving the assembly of these high-order CRP_MTB_ oligomers on DNA. We hypothesize that the formation of oligomers could either be attributed to nonspecific DNA interactions or occur through free proteins anchoring themselves to pre-existing specific CRP_MTB_–DNA complexes.

**Figure 5:**
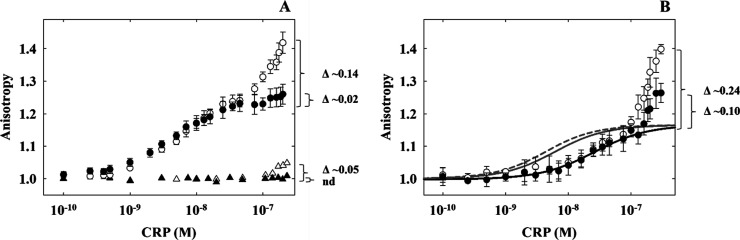
“Scramble” DNA and serC32 “half-site” promoter binding. A. CRP_MTB_ wildtype and “Scramble” (32 bp) anisotropy with (filled triangles) and without (empty triangles) cAMP. The CRP_MTB_ wildtype data for serC32 full-site with (filled circles) and without (empty circles) cAMP (Figure 4) is shown for reference. B. CRP_MTB_ wildtype and serC32 “half-site” anisotropy with (filled circles) and without (empty circles) cAMP. The solid and dashed black lines represent the fits using eq 8 with and without cAMP, respectively. Modeled data using the fitted *K*_*DNA*_ and *k*_NSP-cAMP_ values from serC32 full-site data and *A*_P-DNA_ from serC32 “half-site” data with (solid gray lines) and without (dashed gray lines) cAMP are shown for reference.

To dissect the contributions of these two possible mechanisms, we analyzed previously published data on CRP_MTB_’s binding data for a scramble DNA sequence, which only allows nonspecific binding.^27^ This comparison aimed to differentiate nonspecific DNA interactions from targeted binding to the serC promoter. The analysis revealed that, without cAMP, CRP_MTB_ minimally interacts with the scrambled DNA at high protein concentrations as observed at the end of the titration (Figure 5A). In the presence of cAMP, there is no change in the anisotropy signal, suggesting that the cAMP–CRP_MTB_ complex does not interact with the scrambled DNA, possibly due to a reduction or complete loss of nonspecific interactions. When comparing the relative anisotropy scale for nonspecific interactions in the absence of cAMP to the full serC site, we observe a significant rise in the anisotropy signal (Figure 5A). This increase indicates the formation of oligomers that cannot be the result of nonspecific DNA binding. Moreover, in a previous study using a 20-bp serC promoter—which has 1 bp outside the CRP_MTB_–DNA contact surface, thereby minimizing nonspecific DNA interactions—we showed the formation of oligomers exceeding a one-to-one molar ratio of CRP_MTB_ to DNA.^27^ Altogether, it is more likely that apo CRP_MTB_ bound to the specific serC site serves as a nucleation point for the formation of high-order oligomers through the association of other free apo CRP_MTB_ proteins.

### Role of DNA in CRP_MTB_ Allostery

Next, we investigated whether cAMP solely inhibits the binding of free proteins to preformed CRP_MTB_-DNA complexes, or if DNA also plays a contributory role. To explore this, we conducted DNA binding experiments using a modified serC sequence with half of its binding site scrambled, creating a ’half-site’ scenario that permits only one protomer of the CRP_MTB_ homodimer to form specific interactions (Figure 5B). The findings revealed a significant reduction in *K*_DNA_ and *K*_DNA-cAMP_ for the serC half-site, both decreasing 5-fold to (4.3 ± 0.1) × 10^7^ M^–1^ and (4.0 ± 0.1) × 10^7^ M^–1^, respectively. In the absence of cAMP, the formation of high-order oligomers occurred at comparable CRP_MTB_ concentrations as the full serC site. Intriguingly, while the addition of cAMP diminished the assembly of high-order oligomers, it did not reduce them to the extent observed with the intact serC sequence (Figure 5B). This result suggests that, even in the presence of excess cAMP, proteins continue to bind to preformed CRP_MTB_–DNA complexes, implying that cAMP alone cannot prevent proteins from associating to these complexes. Effective dissociation seems to require both cAMP–CRP_MTB_ and specific interactions with the two cognate sites within the DNA promoter.

### Mutations Affect CRP_MTB_-DNA Oligomer Formation but Not DNA Binding Affinity

Our investigations with CRP_MTB_ wildtype indicate that cAMP exerts its most significant effect when the full site of the serC promoter is available. Additionally, the full site offers the strongest specific binding affinities. Therefore, to assess the potential allosteric effects of structurally homologous mutations on either specific binding or oligomer formation, we conducted measurements using the serC full-site.

Although equivalent mutations in CRP_Ecoli_ significantly impact specific DNA binding,^28,29,34^ our findings show that for all five CRP_MTB_ mutants, the binding affinities to the serC promoter were comparable to wildtype, regardless of cAMP presence (Figure 4C, Table 3). These results suggest that the mutations do not interfere with the coupling between the CBD and DBD in CRP_MTB_ in terms of specific interactions with DNA promoter sequences.

**Table 3:** DNA Binding Affinities and Δ*G*_cAMP_’s of CRP_MTB_ Wildtype and Mutants^a^

CRP_MTB_ protein	*K*_DNA_	*K*_DNA-cAMP_	Δ*G*_cAMP_
wildtype	1.87 ± 0.01	2.81 ± 0.01	0.59
A61H	4.03 ± 0.08	2.95 ± 0.05	0.25
A61I	2.71 ± 0.02	3.57 ± 0.03	0.34
D63H	4.53 ± 0.04	4.66 ± 0.06	0.59
T70F	4.42 ± 0.04	2.75 ± 0.02	0.00
G148S	2.43 ± 0.02	1.93 ± 0.02	0.10

a CRP_MTB_, cAMP-receptor protein from *Mycobacterium tuberculosis*; *K*_DNA_, specific DNA-binding affinity in the apo state; *K*_DNA-cAMP_, specific DNA-binding affinity in the doubly cAMP-bound state; Δ*G*_cAMP,_ Gibb’s free energy associated with the stabilization of high-order oligomers as a function of cAMP. The error corresponds to the SD from fitted parameters as described in Materials and Methods. The units of *K*_DNA_ and *K*_DNA-cAMP_ are 10^8^ M^–1^. The units of Δ*G*_cAMP_ are 10^8^ kcal·M^–1^.

However, the binding transition representing the formation of high-order CRP_MTB_ oligomers on DNA did show differences between CRP_MTB_ wildtype and mutants. To quantify these differences, we estimated how much cAMP binding reduces the formation of oligomers (defined as Δ*G*_cAMP_ in kcal·mol^–1^) using a threshold-concentration difference for the formation of oligomers with and without the cyclic nucleotide (Figure 4A; Materials and Methods). For CRP_MTB_ wildtype, oligomers form at protein concentrations of 1.5 × 10^7^ M^–1^ and 0.6 × 10^7^ M^–1^ in 0 mM and 1 mM cAMP conditions, respectively (Figure 4A), which translates into Δ*G*_cAMP_ = 0.59 kcal·mol^–1^. Of all the mutants, only D63H retains identical effects of cAMP compared to wildtype. A61H and A61I also reduce oligomer formation upon cAMP binding, albeit to a lesser extent than D63H and wildtype. However, the effect of cAMP to reduce oligomers for T70F and G148S is lost, as indicated by indistinguishable DNA binding isotherms across the entire protein concentration range with and without cAMP (Figure 4C, Table 3).

## Discussion

The cAMP-dependent activation mechanism of CRP from *E. coli* is well-characterized, however, there is limited research into how its homologue from *M. tuberculosis* (MTB) is allosterically regulated by the same cyclic nucleotide. Here we take advantage of the structural similarity between CRP_Ecoli_ and CRP_MTB_ to identify conserved allosteric hotspots, and test if structural homology can be a valuable predictor of allosteric homology.

### Mutational Effects on cAMP Binding

The cyclic nucleotide-binding domain is a widely found and structurally conserved signaling module capable of regulating various proteins through allosteric mechanisms.^35^ This includes proteins like kinases,^36^ bacterial transcription factors,^27,32^ ion channels,^37^ and EPAC proteins.^38,39^ Despite its structural conservation, CBD can propagate allostery across different biological environments, as seen in CRP in *E. coli* and *M. tuberculosis*. While *E. coli* thrives in diverse environments, including both anaerobic and aerobic conditions,^40^*M. tuberculosis* requires oxygen for growth and divides slowly.^41^

**Figure 6:**
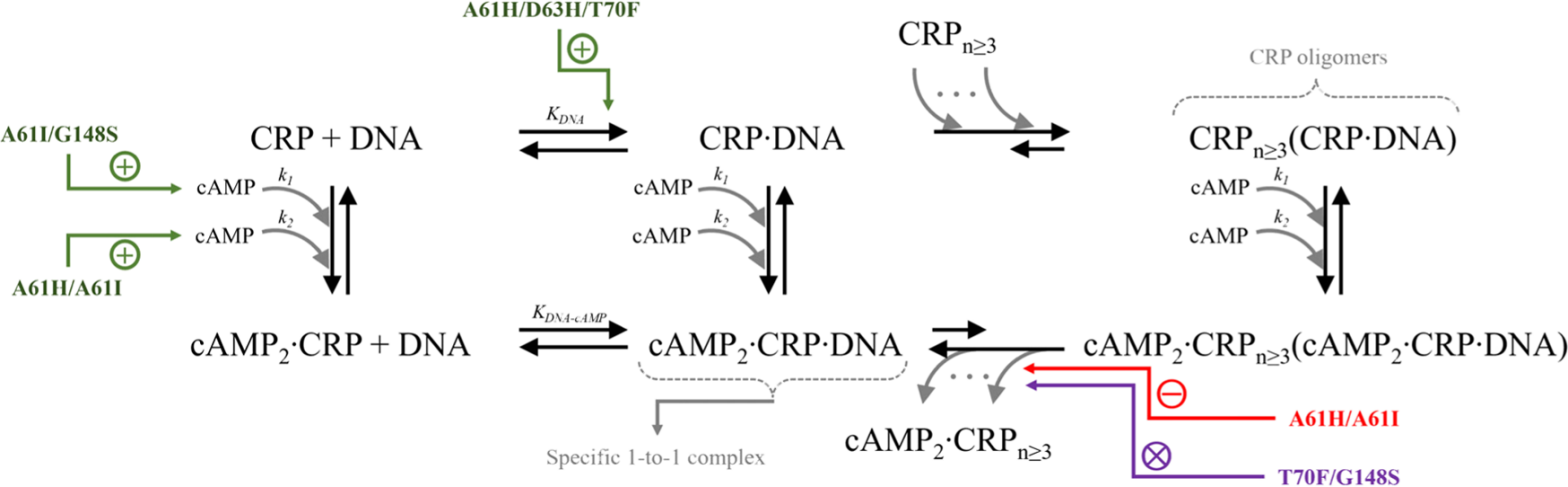
Summary of structurally homologous mutant allosteric effects. The cAMP and DNA binding pathways for CRP_MTB_ wildtype are shown in black with annotations for cAMP binding, specific DNA binding (1-to1 complex), and oligomer formation on DNA. The effect of mutations is indicated in the pathway where their effects were ∼2-fold compared to wildtype. Green “+” indicates an increase in binding affinity for cAMP or DNA. Red “-” indicates a decrease in Δ*G*_cAMP_ and purple “x” indicates a Δ*G*_cAMP_ value near zero, indicating a reduction or complete loss in the ability to reduce oligomers as a function of cAMP, respectively.

Although the structural difference between the cAMP-bound forms of CRP in *E. coli* and *M. tuberculosis* is minimal, the mutational effects of functionally relevant residues for cAMP binding vary (Figure 6). For instance, mutations like G148S in CRP_MTB_ increase negative cooperativity between the cAMP binding sites (Figure 3) whereas the equivalent mutation in CRP_Ecoli_, G141S, increases cooperativity by more than 100-fold.^29^ T70F in CRP_MTB_ has a minimal effect on cAMP binding (Figure 3), yet S62F in CRP_Ecoli_ leads to large negative cooperativity.^28^ Additionally, mutations in the β4/β5 loop of CRP_MTB_ like A61H or D63H have different effects, with A61H increasing cooperativity similar to CRP_Ecoli_ D53H (Figure 3).^28^

Given the established linkage between protein stability and function,^42^ changes in cAMP binding affinity and cooperativity may arise from small changes in stability. For example, the A61H and G148S mutations that impact cAMP cooperativity in CRP_MTB_, have lower global protein stabilities (Figure 2C,D), which is similar to the destabilizing effect of D53H mutation seen for CRP_Ecoli_.^43^ While unfolding free energies for G141S from CRP_Ecoli_ are unavailable, the constitutively active mutants G141K and G141Q lower the free energy of dimer association.^34^ This suggests that the effects of G148S on cAMP binding may also result from population shifts due to decreased stability of CRP_MTB_. Overall, the subtle structural differences observed in the CBD of CRP_MTB_ and CRP_Ecoli_ highlight the significance of structural stability in proteins with conserved CBD, and indicate that differences in cAMP binding and cooperativity cannot be solely attributed to their native structures, mirroring observations in other proteins.^44−46^

### cAMP-Mediated Allostery in CRP_MTB_ is Not Coupled to Specific DNA Interactions

All CRP_MTB_ mutants studied here have minimal impact on specific protein–DNA interactions (Figure 4 and Table 3) where A61H, D63H, and T70F increase by a factor of ∼2 the affinity for the serC promoter in the absence of cAMP (Figure 6). This suggests that mutational effects observed in CRP_Ecoli_ are not preserved. Depending on the CRP_Ecoli_ mutation, DNA affinity to the *lac26* promoter can vary by 10–15-fold.^29,34,47,48^ NMR studies on CRP_Ecoli_ show that despite global rigidification upon cAMP and DNA binding,^29^ certain mutations still achieve specific DNA binding through perturbations to local dynamics and stability.^28,29,48,49^ These effects emphasize that allostery in CRP_Ecoli_ is intimately related to changes in protein dynamics. It can then be expected that changes to protein stability could affect allosteric mechanisms related to DNA binding in CRP_MTB_. However, the mutations A61H and G148S show no effects on DNA binding affinity to the specific site (Figure 4 and Table 3) despite both of them having decreased thermodynamic stability of secondary and tertiary structures (Figure 2C,D and Table 1). Therefore, one may conclude that small perturbations to protein stability outside the DNA binding domain cannot augment specific DNA binding affinity. In other words, the DNA binding domains of CRP_MTB_ might already be optimally configured for specific DNA binding at the promoter site. For instance, the orientation of DBD of CRP_MTB_ in the apo state is similar to the cAMP-bound structure of CRP_Ecoli_ as seen in the structural comparison in Figure 1.

### Mutational Effects on High-order CRP_MTB_ Oligomers on DNA

The Δ*G*_cAMP_ values estimate the free energy associated with the ability of cAMP to reduce higher-order CRP_MTB_ oligomers on DNA. Larger values indicate a stronger reduction effect (Figure 4A). Δ*G*_cAMP_ values in Table 3 reveal that, except for D63H, all mutations weaken the ability of cAMP to reduce the formation of these oligomers compared to CRP_MTB_ wildtype (Figure 6). However, the origins of these mutational effects differ, as Δ*G*_cAMP_ depends on oligomer formation in both the apo and cAMP-bound conformations. In the apo conformation, A61H, T70F, and G148S exhibit a reduced tendency to form oligomers—indicated by oligomers forming only at higher protein concentrations compared to the apo wildtype. In contrast, D63H displays an increased tendency to form oligomers, while A61I shows no effect (Supplementary Table S1). In the cAMP-bound conformation, all mutations except for A61H exhibit a much higher tendency to form oligomers compared to wildtype. Consequently, the net effect that is represented by Δ*G*_cAMP_, indicates the ability of cAMP to reduce higher-order CRP_MTB_ oligomers on DNA is diminished for A61H and A61I and is entirely lost for T70F and G148S (Figure 6).

### Possible Biological Role of High-order Oligomerization in CRP_MTB_

We provide evidence that the formation of CRP_MTB_ oligomers on DNA is primarily due to proteins binding to preformed, one-to-one CRP_MTB_-DNA complexes. While determining the exact geometry and interaction sites of these complexes is out of the scope of the current work, we speculate that the proteins are possibly associated with each other along the *y*-axis opposite from the central helices. This speculation is supported by the PDB: 3I54 crystal structure for cAMP-bound CRP_MTB_, which reveals two dimers associated with each other at this surface.^18^ While these observations may be influenced by crystal packing effects, they suggest that the protein exhibits favorable interactions in this region.

Additionally, previous studies have shown that the activating region 1 (AR1) is similarly structured in other CRP complexes, including CRP_Ecoli_ in complex with DNA and RNAP, reinforcing the biological relevance of this surface for protein–protein interactions. Specifically, the surface-exposed β-loop (residues 156–164) in the AR1 region has been demonstrated to interact with the α-carboxy-terminal domain of RNA polymerase,^50−53^ which is crucial in the mechanism of class I promoters. This suggests that the AR1 may play a significant role in oligomerization and functional interactions *in vivo*. Therefore, this region in CRP_MTB_ could facilitate favorable protein–protein interactions, leading to the formation of high-order oligomers. The biological significance of oligomerization in CRP_MTB_ may be to block RNAP from binding the AR1 region when cAMP levels are low. In this conformation, CRP_MTB_ would be bound to specific DNA sequences along with other CRP_MTB_ proteins, serving as scaffolding for the protein already attached to the site. When cAMP concentration increases, the oligomers would dissociate, exposing the AR1 region of CRP_MTB_ and promoting RNAP association for transcription.

## Conclusions

This study explores the factors contributing to the differential allosteric regulation between CRP_Ecoli_ and CRP_MTB_. Our biophysical and functional studies reveal that structural homology and mutagenesis, based on the well-characterized CRP_Ecoli_, did not reliably identify allosteric hotspots in CRP_MTB_.

In CRP_MTB_, amino acids A61, D63, and G148 are among the most conserved residues within actinobacteria. However, T70 is only found in 4% of actinobacteria, while hydrophobic side chains (Ala, Leu, Ile, and Val) are found in approximately 75%. In CRP_Ecoli_, structurally homologous residues D53 and S62 are among the most conserved in proteobacteria, but G141 is much less frequently observed, with only 6% conservation (Supplementary Figure S1).

The similarities and differences between actinobacteria and proteobacteria suggest that the allosteric behavior in CRP_MTB_ and CRP_Ecoli_ is likely determined not by individual residues but by a minimal set of residues or networks. Identifying these networks will require further exploration of protein properties, incorporating not only evolutionary covariance of residues^55,56^ but also additional approaches based on thermodynamic and dynamic analyses of coupled interactions.^57,58^

## Supporting Information Available

“Supplementary Table S1” contains parameters used for the determination of values reported in Table 3; “Supplementary Figure S1” shows the conservation of residues studied between CRP_MTB_ and actinobacteria, and between CRP_Ecoli_ and proteobacteria (PDF)
Excel file contain all the raw data obtained in this study (XLSX)

## Abbreviations

CRP: cAMP receptor protein
MTB: *Mycobacterium tuberculosis*
*E. coli*: *Escherichia coli*
CBD: cyclic nucleotide binding domain
DBD: DNA binding domain
ANS: 8-anilino-1-napthalenesulfonic acid
