# Correction to “Identifying Allosteric Hotspots in Mycobacterium tuberculosis cAMP Receptor Protein through Structural Homology”

**DOI:** 10.1021/acs.biochem.5c00102

**Published:** 2025-06-05

**Authors:** Stephen P. Dokas, Daniel K. Taylor, Lydia L. Good, Sanuja Mohanaraj, Anna Park, Rodrigo A. Maillard

**Affiliations:** † Department of Chemistry, Georgetown University, Washington, District of Columbia 20057, United States; ‡ Institute of Soft Matter Synthesis and Metrology, Georgetown University, Washington, District of Columbia 20057, United States

The correction includes the addition of one author, Anna Park, whose work on A61G mutation was not recognized in the original publication. No additional corrections are being made. The authorship change is reflected in the authorship of this Correction.

