# Supplementary material for: Identifying Allosteric Hotspots in Mycobacterium tuberculosis cAMP Receptor Protein through Structural Homology
Source: Biochemistry. 2025 Jan 31;64(4):801–11. doi: 10.1021/acs.biochem.4c00723 (PMC11840924; doi:10.1021/acs.biochem.4c00723)
Supplement: Supplementary file 1 — bi4c00723_si_001.pdf [file bi4c00723_si_001.pdf]

SUPPORTING INFORMATION

Identifying allosteric hotspots in *Mycobacterium tuberculosis* cAMP receptor protein through structural homology

*Stephen P. Dokas[1], Daniel K. Taylor[1], Lydia L. Good[1], Sanuja Mohanaraj[1], Rodrigo A. Maillard[1]**

[1]Department of Chemistry, Georgetown University, Washington, DC 20057, USA

Supplementary Table 1. Values used to determine ΔG

	[CRP] boundaries		'*k* apparent'		ΔG	
**CRP_{MTB} protein**	**apo**	**cAMP-bound**	**apo**	**cAMP-bound**	**apo**	**cAMP-bound**
wildtype	65	175	0.15	0.06	-9.799	-9.213
A61H	105	160	0.09	0.06	-9.515	-9.266
A61I	56	100	0.18	0.10	-9.887	-9.544
D63H	27.5	75	0.36	0.13	-10.308	-9.714
T70F	100	100	0.10	0.10	-9.544	-9.544
G148S	85	100	0.12	0.10	-9.640	-9.544

CRP_{MTB}, cAMP-receptor protein from *Mycobacterium tuberculosis;* CRP boundaries, protein concentrations used to determine the boundary for the affinity of formation of oligomers; '*k* apparent', apparent affinities for boundaries for the affinity of formation of oligomers using $k = 1/[CRP]$; ΔG, Gibb's free energy associated with the '*k* apparent' using Equation 4. The units of [CRP] are in 10^{-9} M. The units of '*k* apparent' are 10^{8} M^{-1}. The units of ΔG are 10^{8} kcal·M^{-1}.

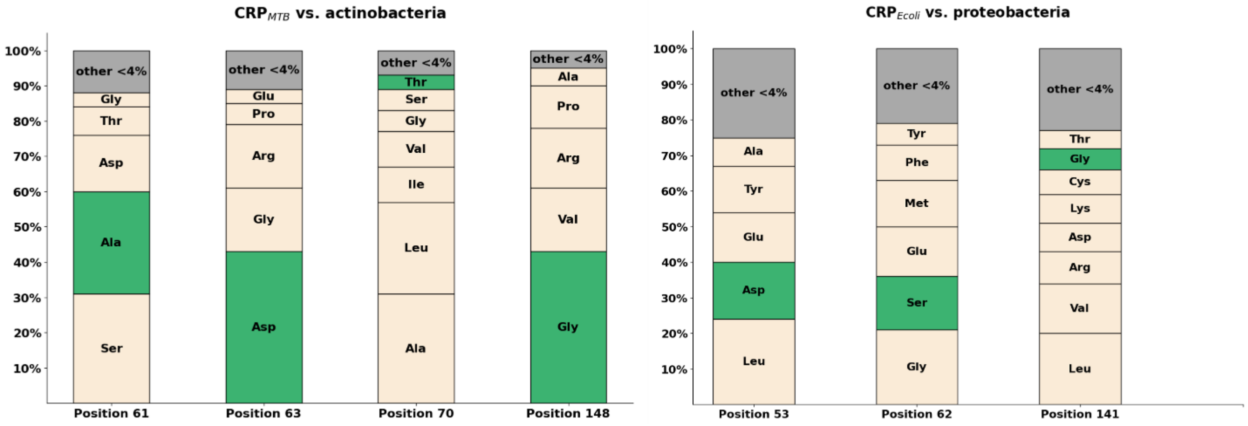

Supplementary Figure 1. Sequence conservation of residues studied in CRP_{MTB} against actinobacteria and residues in CRP_{Ecoli} against proteobacteria are shown on the left and right, respectively. The wildtype residue identities are indicated in green; all residue identities exceeding greater than 4% frequency are indicated in tan; residue identities with less than 4% frequency are grouped in "other" and are indicated in gray. The organisms and their accession code for which this was derived is found in Source Data.

Corresponding Author

*Rodrigo A. Maillard. Department of Chemistry, The Graduate School of Arts & Sciences, Georgetown University, Washington, DC 20057, USA; Email: rodrigo.maillard@georgetown.edu
